# Can cone beam CT predict the hardness of interradicular cortical bone?

**DOI:** 10.1186/1746-160X-10-12

**Published:** 2014-04-15

**Authors:** Tamar Brosh, Bereznyak-Elias Yekaterina, Raphael Pilo, Nir Shpack, Silvia Geron

**Affiliations:** 1 Department of Oral Biology, The Goldschleger School of Dental Medicine, Tel Aviv University, Tel Aviv 69978, Israel; 2 Department of Orthodontics, The Goldschleger School of Dental Medicine, Tel Aviv University, Tel Aviv 69978, Israel; 3 Department of Oral Rehabilitation, The Goldschleger School of Dental Medicine, Tel Aviv University, Tel Aviv 69978, Israel

**Keywords:** Density, Microhardness, Mandible, Maxilla, Interradicular

## Abstract

**Objectives:**

Orthodontic mini implants can be inserted at the interradicular site. The bone quality at this site may affect the stability and anchorage of the implant. Bone density is clinically evaluated by Hounsfield units (HU) obtained from cone beam CT (CBCT). The objective of this study was to determine the correlations between HU, microhardness and cortical bone thickness of interradicular site at various segments (anterior/posterior) and aspects (buccal/lingual) of both jaws in a swine model.

**Materials and methods:**

Eight mandible and maxilla swine bones were scanned by CBCT. The HU and thickness of the above-mentioned sites were determined. Then, a Knoop microhardness test was applied and the Knoop Hardness Number was obtained (KHN).

**Results:**

The mandible parameters spread over a wider range than the maxilla. The buccal aspect of the maxilla had higher HU and KHN values than the mandible. The lingual aspect of the mandible had higher KHN values than the maxilla. Posterior segments had higher HU and KHN values. The thickness of the alveolar cortical bone was greater in the maxilla than in the mandible. Correlations were found between HU and KHN for 3 of the 4 sites (anterior or posterior, buccal or lingual) of the mandible only. No correlations were found for the maxilla. Upon pooling the HU and KHN data for the whole jaw, correlation was found for the maxilla as well.

**Conclusions:**

Relying on HU values as a predictor of cortical bone hardness should be considered with caution.

## Introduction

Currently, orthodontic mini implants (OMIs) are widely used as anchorage support during orthodontic treatment [1]. These implants are inserted with a minor surgical procedure and loaded immediately. Force magnitudes and directions applied on the OMIs vary according to the desired treatment plan [2]. Their success, however, depends on proper initial mechanical stability and loading capabilities [3]. OMIs can be inserted into the palate and occasionally into the bone located between the roots of various teeth in the maxilla or mandible of both jaws in either the buccal or the lingual aspects.

In the early stages of an orthodontic treatment using mini implants, osseointegration does not exist and only screw tightening to the alveolar bone holds the implant in place. Thus, bone quality is the primary factor influencing the initial stability. The mechanical stability of OMIs is mainly studied by measuring either the insertion torque [4] or the force needed for pulling the OMI from the bone along the path of insertion [5].

Clinically, information obtained by computed tomography (CT) or cone beam CT (CBCT) is used for analyzing the quality and quantity of bone. Many studies have investigated the outcomes of these scanning methods in relation to OMIs. The Hounsfield Unit (HU) is the parameter defined by CT for quantifying bone quality [6]. This parameter is based on the linear attenuation of the radiodensity of materials relative to water (HU = 0). Regarding the human jaws at various heights of the alveolar crest characterizing the interradicular zones, the buccal mandibular posterior sites showed statistically greater bone densities compared to the related maxilla-sites, while the differences in the anterior areas were not significantly different [7]. The correlation between CT or CBCT and the success rate of OMIs in the alveolar bone was determined from a literature review [8]. It was concluded that the posterior area is the best site for inserting mini implants, with no preference for the left or right side.

The thickness of the cortical bone in the interradicular zone is important for the success of OMIs [9,10]. The necessary thickness of the cortical plate of the alveolar bone and the distances from the roots of the teeth in the interradicular areas were determined for OMI placement [11-14]. The buccal alveolar bone between the second premolar and the first molar in the maxilla and the interradicular spaces from the first premolar to the second molar in the mandible were found to be adequate for OMIs [11]. The interradicular space of molars was suggested as a favorable site for OMIs [12]. In this study it was found that the lingual mandibular cortical thickness was greater than the buccal one, while the maxillary buccal cortical thickness was less than the mandibular in the molar zones. In another study, the thickness of the mandibular buccal plate was higher than the lingual one [13]. That is, differences in cortical bone thickness between and within regions of the jaws are evident [14,15]. Nevertheless, a lower threshold of 1 mm was suggested for cortical bone thickness [16]. Although the thickness of the bone is important, the mechanical quality of the bone should also be assessed, but this can only be accomplished ex vivo. Thus, the relationship between the mechanical properties and HU data may provide insight into the clinically provided CT data.

A reliable tool for determining the mechanical property of bone for such a small interradicular site is the microhardness test [17-19]. However, the correlation between microhardness data and HU in these sites has never been determined. The aim of this study was to measure the HU, the microhardness and the cortical bone thickness at various interradicular segments (anterior or posterior) and aspects (buccal or lingual) of both jaws in a swine model and to determine the correlations between these parameters.

## Materials and methods

**Figure 1:**
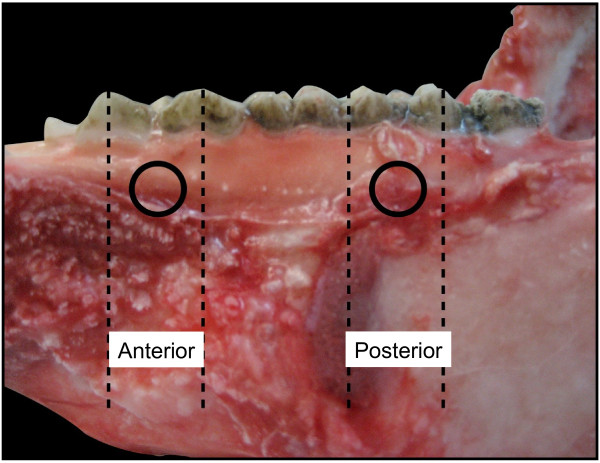
**Buccal view of the lower jaw of the swine model.** Dotted lines indicate the anterior and posterior interradicular segments. Circles indicate the specific buccal anterior and posterior test sites.

Eight maxillae and 8 mandibles of 6-month-old (90–100 Kg) domestic swine (Lahav, C.R.O, Israel) were examined. The animals were raised as herd for meat. After slaughter, the jaws were extracted, carefully cleaned of soft tissues and kept frozen (-20°) while wrapped in gauze with saline. Four interradicular sites in each jaw, representing possible sites for OMI insertion, were selected for testing the bone density (HU), cortical bone thickness and microhardness (Figure 1). The 4 sites were divided into 2 segments: anterior (A), between the second and third premolar, and posterior (P), between the first and second molar. The A or P segments were further subdivided into 2 aspects: buccal (B), taken from the right side, and lingual (L), taken from the left side, of each jaw. The latest was done in order to allow further parallel planes cuttings for the microhardness tests (see below).

### CBCT scans

3-D dental CT radiographs were performed on each jaw via cone beam x-ray using an anode with 2–10 mA and a cathode with 75 KV (Picasso-trio, Korea, 2007). The digital files were copied to a CD-ROM and downloaded to a personal computer for analysis. Bone density (HU) and interradicular alveolar bone thickness (mm) were analyzed using an appropriate program (EzImplant, Korea, 2007). The coronary area of each of the above-mentioned sites was viewed on the computer screen. Analysis was performed at the maximal alveolar bone thickness for that site. The thickness in mm and the maximal HU value were registered along a path of 1.4 mm. These measurements were repeated 4 times, and the mean values for the thickness and density were calculated.

### Microhardness test

The jaws were defrosted at room temperature, and the sites that had been analyzed using the CBCT scan were marked. Each jaw was dissected sagittally between the incisors for 2 halves, left and right, using a saw. Then, coronary cuts were performed, producing 4 segments (2 anterior and 2 posterior) of 10 mm mesio-distal blocks, including the areas to be tested. Each block was prepared for microhardness testing at the buccal or lingual aspect. Two parallel planes were cut in each block using a disk (Isomet Plus, Buehler, Lake Bluff, Illinois, USA). For example, if the buccal aspect had to be measured, a layer that was 0.3 mm thick was removed from that side at the maximal alveolar bone thickness. Then, an additional parallel cut was made approximately 10 mm lingually. Finally, the surface to be tested was polished (Ecomet, Buehler, Lake Bluff, IL, USA). Three polishing papers with decreasing grit (No. 320, 400, and 1200) (WS-Flex, waterproof abrasive paper, Hermes, G-18) followed by 2 diamond pastes (6 and 1 micron) completed the preparation of the specimens for microhardness testing.

A Knoop microhardness test (presented as Knoop Hardness Number, KHN) was performed with an applied force of 100 g for 10 s (Microhardness tester, model DMH-2, Matsuzawa Seiki, Tokyo, Japan). Each site was indented 4 times with 75 microns between indentations, where the long diagonal of the indenter was oriented mesio-distally. The mean KHN value was calculated.

### Statistics

Analysis of variance (ANOVA) with repeated measures and 2 within-subject factors - buccal versus lingual aspects and premolar versus molar segments - was performed for verifying the differences between the sites for each jaw mandible and maxilla (SPSS Ver. 15.0). A Pearson correlation test between the parameters was applied, and significant differences were considered as p < 0.05.

## Results

**Figure 2:**
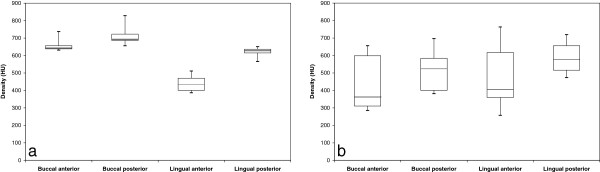
**Box diagrams of HU for the (a) maxilla and (b) mandible.** Boxes represent the 1^st^ and 3^rd^ quartiles, the band inside is the median and the whiskers are the minimum and maximum values of all of the data.

**Figure 3:**
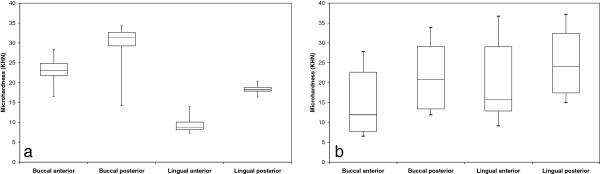
**Box diagrams of microhardness for the (a) maxilla and (b) mandible.** Boxes represent the 1^st^ and 3^rd^ quartiles, the band inside is the median and the whiskers are the minimum and maximum values of all of the data.

**Figure 4:**
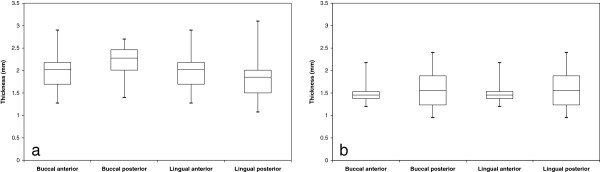
**Box diagrams of cortical bone thickness for the (a) maxilla and (b) mandible.** Boxes represent the 1^st^ and 3^rd^ quartiles, the band inside is the median and the whiskers are the minimum and maximum values of all of the data.

Figures 2, 3 and 4 present box plot diagrams of the density (HU), microhardness (KHN) and thickness values, respectively, for all 4 sites of the 2 jaws. For all of the parameters, the variances were higher in the mandible compared to the maxilla. This result is indicated by the larger box plots and higher standard deviations.

### Density

The HU values varied between 257 and 828. The mean values (SD) for the mandible were: B-A: 439.0 ± 159.5, B-P 516.2 ± 117.9, L-A 469.5 ± 177.5, L-P 586.2 ± 87.0 and for the maxilla: B-A 656.5 ± 35.0, B-P 715.3 ± 56.8, L-A 439.4 ± 45.7, L-P 619.0 ± 29.9.

Between jaws: The HU values in the buccal aspect of the maxilla (in both the A and P segments) were 44% higher compared to the mandible (p = 0.006) (Figure 2a, b).

Maxilla: The buccal aspect (both A and P) had 30% higher HU values compared to the lingual aspect (p = 0.003). The posterior segments had 22% higher HU values than the anterior ones in both the buccal and lingual aspects (p = 0.015). An interaction exists between the aspects (B and L) and the segments (A and P) (p = 0.003), indicating that the increase in HU values from anterior to posterior is much higher in the lingual aspect compared to the buccal aspect (Figure 2a).

Mandible: No significant differences were found between the buccal and lingual HU values (p = 0.554). The posterior segments of the mandible had 21% higher HU values compared to the anterior specimens (p = 0.012); however, this finding is attributed to the posterior lingual site, which was 25% higher than the anterior lingual site (p = 0.005). No interaction was found between the aspects (B and L) or the segments (A and P) (p = 0.613) (Figure 2b).

### Microhardness

The KHN values varied between 6.55 and 37.2. The mean values (SD) for the mandible were: B-A: 15.01 ± 8.73, B-P 21.73 ± 8.85, L-A 20.20 ± 10.38, L-P 24.92 ± 8.78 and for the maxilla: B-A 23.2 ± 3.60, B-P 29.38 ± 6.34, L-A 9.49 ± 2.25, L-P 18.29 ± 1.20.

Between jaws: The maxilla at the buccal anterior zone had 55% higher KHN values compared to the respective value of the mandible (p = 0.036); on the other hand, at the lingual aspect, the values were 2-fold higher in the mandible compared to the maxilla (p = 0.022) (Figure 3a, b).

Maxilla: The buccal aspect had 89% higher KHN values compared to the lingual aspect (p = 0.0013). The posterior segment had 46% higher values than the anterior in both the buccal and lingual aspects (p < 0.001). No interaction existed between the aspects (B and L) and the segments (A and P) (p = 0.138) (Figure 3a).

Mandible: No significant differences were found between the buccal and lingual aspects of the mandible (p = 0.505). The posterior segments had 33% higher KHN values compared to the anterior segments (p < 0.001). No interaction existed in the mandible between the aspects (B and L) and the segments (A and P) (p = 0.532) (Figure 3b).

### Thickness

Cortical bone thickness varied between 0.95 mm and 3.1 mm. The mean values (SD) for the mandible were: B-A: 1.50 ± 0.30, B-P 1.58 ± 0.48, L-A 1.21 ± 0.23, L-P 1.75 ± 0.40 and for the maxilla: B-A 1.99 ± 0.50, B-P 2.15 ± 0.48, L-A 2.05 ± 0.37, L-P 1.85 ± 0.63.Between jaws: The thickness of the alveolar cortical bone was 33% greater in the maxilla than in the mandible (p < 0.038) (Figure 4a, b).

Maxilla: No significant differences in thickness were found between the buccal and lingual aspects (p = 0.264) or between the segments (A and P) (p = 0.936). A borderline interaction existed between the aspects (B and L) and segments (A and P) (p = 0.069) (Figure 4a). From the anterior to the posterior zone, there is an increase in the thickness in the buccal aspect and a decrease in the lingual aspect.

Mandible: No significant differences were found between the buccal and lingual aspects (p = 0.649). The posterior segments had 23% higher thickness compared to the anterior ones (p = 0.038); however, this difference was significant (32%, p = 0.012) in the lingual aspect only. An interaction existed in the mandible between the aspects (B and L) and the segments (A and P) (p = 0.017) (Figure 4b).

### Correlations

Correlations were applied separately between parameters for each site. No correlations between microhardness and density were found in the maxilla. However, in 3 of the 4 sites in the mandible, significantly high correlations were found in the anterior and posterior buccal sites (R = 0.942, p < 0.001 and R = 0.898, p < 0.002, respectively) and in the lingual anterior site (R = 0.974, p < 0.001). A correlation between density and cortical bone thickness was found in the posterior lingual site of the mandible only (R = 0.831, p < 0.011). No correlations were found between cortical bone thickness and microhardness.

## Discussion

**Figure 5:**
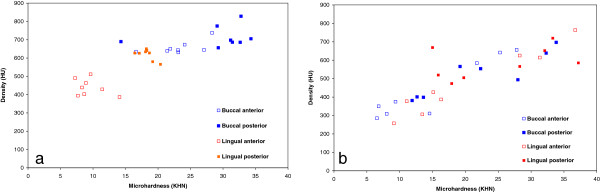
HU vs. microhardness for the each jaw including all tested sites (a) maxilla and (b) mandible.

The hypothesis of the current study - that a correlation exists between the bone density as obtained from CBCT scan and the mechanical property of the alveolar bone as determined by a microhardness test was verified only for the mandible. High correlations were found in 3 of the lower jaw sites (except for the posterior lingual site) and in none of the sites for the maxilla. The cluster of values in the maxilla, when analyzing each site separately, does not indicate that significant correlations exist between the density and microhardness for each zone separately. However, upon pooling the HU and hardness data for the whole jaw (Figure 5), a significant correlation between these parameters was obtained for the maxilla as well (Maxilla Figure 5a: R = 0.834, mandible Figure 5b: R = 0.866, p < 0.001). This is because higher HU and KHN values characterize the buccal posterior site and lower HU and KHN values characterize the lingual anterior site in the maxilla. Our results partially agree with a previous study that used a 3-point bending test and found a weak correlation between modulus values and bone density values obtained by CT of the mandible. They concluded that CT scans would not be sufficient for accurately predicting the mechanical properties of bone [20]. The disadvantage of that research is that large specimens (20 mm in length) are needed for a 3-point bending test, and the bone in the interradicular space is not adequate for such a test. By using the microhardness test, as in the current study, measuring the mechanical properties of the cortical bone in this site is possible. In connection to our study, the mineral density of cortical bone after the placement of OMIs in cadavers was related to the hardness and the modulus of elasticity [21]. Moreover, in a study of synthetic bones in which the “cortical layer” was uniform, it was also shown that a significant increase in the insertion torque occurred as the cortical bone density increased [22]. The limitation here is that these artificial materials do not reflect the complexity of the natural cortical bone tissue. When compared to other types of bones, such as the cortical bone in the femoral midshaft, no correlation existed between the bone modulus and the CT number. Thus, the placement of OMIs based on only the HU data should be taken with caution and should be limited to the mandible.

The thickness of cortical bone, usually assessed by CT, has been used to suggest good locations for OMIs [16,23-26]. However, no correlations were found in our study between the thickness of the cortical plate and the other tested parameters, implying that increased thickness is not a good predictor of improved mechanical properties, which agrees with Huja et al., 2005 [5], who reported a weak correlation (R = 0.39) between the pull-out force of OMIs and cortical bone thickness. Cortical bone thickness was related to failure force in a laboratory study of OMIs [21], with an 8 mm implant having a higher success rate compared to a 6 mm implant [26]. Although a minimal cortical thickness of 1 mm is suggested for the placement of OMIs [10], it is recommended that the OMIs be inserted at an incline position [27]. OMI implants are inserted in both cortical and trabecular bone and predictions of the mechanical properties of the trabecular jaw bone based on HU values were reported to be valid for jaws with a thin layer of cortical bone [28]. For jaws with a thicker cortical layer, the prediction of the mechanical properties decreased significantly. Regarding dental implants, the insertion torque was moderately correlated with CT or CBCT [29], implying that the success of OMIs is mainly due to the retention between the screw threads within the bone. Poor quality or an insufficient quantity of available bone may result in a lack of retention [30].

The HU data for both jaws in this study, varied between 257 and 828 with no significant differences between the jaws. Only the buccal aspect of the maxilla had higher HU values. This result is in contrast to a study of human bone that found differences in the densities between jaws, with higher values in the mandible [7]. However, the mean HU values were higher in the posterior areas of both jaws in accordance with previous studies. Nevertheless, the density of the mandible (Figure 2b) has much more variation compared to the maxilla for each zone (Figure 2a). A smaller data range for the maxilla was also found (between 810 and 940 HU) in the alveolar bone [6]. In another study of human bone, HU values at the interradicular sites were higher than 850 HU [7]. The low HU values obtained in this study can be related to the young age of the animals, as previous studies have shown that the densities of adult bones are significantly higher than those of adolescents [15,31].

Comparisons of KHN values between jaws show significant differences in the anterior segment only but these differences were aspect dependent: in the buccal aspect, the values are higher at the maxilla, while in the lingual aspect the values are higher in the mandible. A higher elastic modulus of cortical bone on the lingual side of the dog mandible was also found [32]. In humans, the buccal mandible hardness was found to be higher than in the maxilla [21].

The animal model showed that the thickness of the alveolar bone of the maxilla is greater than in the mandible and that in the mandible, only the buccal posterior site had higher thickness compared to the anterior site. It has to be emphasized that both the anterior and posterior segments in this swine model are actually on the buccal aspect of the animal and not really at the incisors. Nevertheless, variations in cortical thickness of human jaws are known to occur at the interdental sites [9,33,34], and differences are found not only between regions but also within the same region, with cortices of adults being significantly thicker compared to adolescents [12,15].

Microhardness is adequate for measuring mechanical properties of small areas, such as the interradicular sites, but it provides surface information for the tested area only; therefore, it is not representative of the whole cortical bone through its thickness, as provided by the HU value. More studies are needed to determine the relationship between the mechanical properties and the density of the significant, small interradicular spaces.

## Conclusions

Within the limits of the animal investigation presented, the hardness of the interradicular sites is directly related to the bone density obtained via CT only in the mandible. Therefore, caution should be exercised when relying on HU values as a predictor of cortical bone hardness.

## Abbreviations

CT: Computed tomography; CBCT: Cone beam CT; HU: Hounsfield units; KHN: Knoop Hardness Number; OMI: orthodontic mini implants; A: Anterior; P: Posterior; B: Buccal; L: Lingual; ANOVA: Analysis of variance.

## Competing interests

The authors declare that they have no competing interests.

## Authors’ contributions

TB participated in the design of the study and performed the statistical analysis, interpretation of data and writing the manuscript. BEY participated in the design of the study, made all measurements and data acquisition and participating in the statistical analysis. RP have been involved in drafting the manuscript and revised it critically from scientifically point of view. NS have been involved in drafting the manuscript and revised it critically from orthodontic point of view. SG have made substantial contributions to conception and design of the study, interpretation of data and revising the draft of the manuscript. All authors read and approved the final manuscript.
